# Editorial: Bystanders' roles in workplace bullying: impacts and interventions

**DOI:** 10.3389/fpsyg.2026.1763120

**Published:** 2026-03-05

**Authors:** Yariv Itzkovich, Margaret Hodgins, Patricia Mannix-McNamara

**Affiliations:** 1 Department of Sociology and Anthropology, Ariel University, Ariel, Israel; 2 Health Promotion Research Centre, School of Health Sciences, University of Galway, Galway, Ireland; 3 Faculty of Education and Health Sciences, University of Limerick, Limerick, Ireland

**Keywords:** artificial intelligence, bystanders, interventions, observers, workplace bullying

Interpersonal harm remains a pervasive challenge in both higher education and corporate life. Across contexts, individuals must interpret ambiguous social cues, regulate emotional reactions, and decide whether, how, and when to intervene as bystanders of these adversities. Institutions, in turn, structure the opportunities and constraints that shape these judgments. The articles in this Research Topic collectively enrich contemporary scholarship by illustrating how psychological processes and institutional systems jointly influence responses to interpersonal harm, and by demonstrating that these responses cannot be understood through individual psychology alone. Rather than approaching these studies as isolated empirical contributions, this editorial synthesizes the overarching themes that emerge across them. Their convergence provides a coherent picture of the intrapersonal, interpersonal, and structural forces that govern human behavior in the presence of harm.

A central insight across the issue is that observers do not evaluate harmful acts neutrally. Instead, judgments are filtered through personal resources, value systems, and gendered ideologies (e.g., Shi and Zheng, 2021). Following their evaluation, observers' reactions to harmful behavior, whether sexual harassment, exclusion, or informal workplace gossip, were shaped more by the observer's moral orientation and identity than by the objective features of the event. This pattern reflects longstanding empirical evidence that moral judgments arise from intuitive, value-driven processes rather than detached reasoning (Brüggemann et al., 2019) and that gender norms contribute significantly to how observers interpret harassment and aggression (Shi and Zheng, 2021).

Another cross-cutting theme is that empathy, often understood as the foundation of moral responding, is contextually fragile. Individuals reduced their empathic engagement when witnessing social or interpersonal harm. This aligns with contemporary theories of motivated empathy (Zaki, 2014), suggesting that individuals may intentionally down-regulate empathy to avoid personal distress, social cost, or moral conflict. Electroencephalography evidence, showing reduced neural markers of empathy during exposure to social exclusion, strengthens this argument and demonstrates that empathic withdrawal occurs at both the psychological and neurobiological levels. Similar to previous contributions (Itzkovich and Dolev, 2021), the Research Topic, therefore, underscores that empathy is not simply a trait but a resource, influenced by perceived consequences and social context.

While this collective evidence shapes one facet of the overall picture, A unifying insight that emerges from the topic is that interpersonal harm cannot be meaningfully understood through individual psychology alone. Institutional features, hierarchical structures, norms of silence, reporting procedures, cultural expectations, and leadership strongly influence whether individuals recognize harm and take action.

Frameworks adapted within the topic highlight how power asymmetries inhibit intervention, especially in academic contexts where career advancement is contingent on relationships with supervisors. These insights echo research on institutional betrayal (Smith and Freyd, 2014), demonstrating that organizational inaction or complicity can exacerbate harm, suppress reporting, and reinforce cycles of abuse. Across contributions, it became clear that institutional context is not a backdrop but an active mediator of human behavior, shaping the moral calculus that individuals perform when deciding how to respond to harm.

Collectively, the research presented in this issue advocates for a multilevel intervention framework that integrates and aligns individual empowerment, group norm-shaping, and institutional accountability mechanisms. Yet despite this comprehensive approach, empirical evidence reveals a persistent challenge: bystander intervention remains unreliable. While many individuals possess the motivation to intervene, their actions are frequently constrained by emotional self-preservation, ambiguity about appropriate responses, institutional barriers, and concerns about personal or professional repercussions. These limitations suggest that dependence on voluntary human intervention alone is no longer adequate.

This reality underscores the contribution of incorporating artificial intelligence systematically into the detection, prevention, and remediation of interpersonal harm. AI-enabled systems, including automated detection algorithms, behavioral risk assessment tools, and immersive simulation-based training, provide scalable, consistent capabilities for identifying harmful interactions, facilitating timely reporting, and enhancing bystander decision-making in situations where human responses are typically inhibited. Rather than supplanting human discretion, AI functions as a complementary resource that mitigates documented psychological barriers, improves real-time awareness of emerging risks, and establishes clear intervention protocols when individuals are reluctant or unable to act independently.

As workplace harm increasingly manifests through digital channels and complex interpersonal dynamics, AI-augmented frameworks for prevention and intervention constitute an essential evolution in organizational safety infrastructure. We hope these reflections catalyze further research and practical applications toward creating work environments that are both safer and more inclusive.

**Figure 1:**
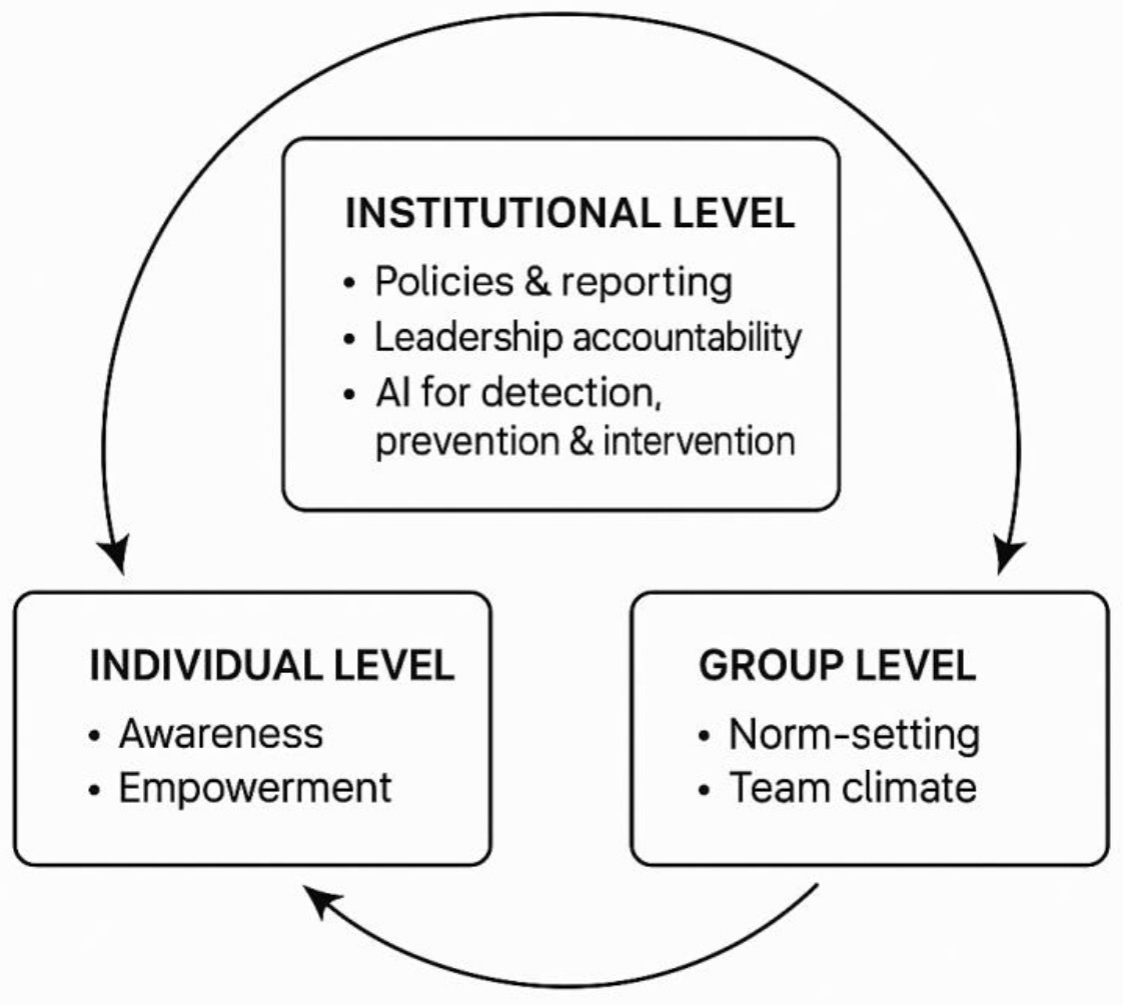
A multilevel intervention framework.

Figure 1 shows embedding AI at the institutional level. While substantial evidence demonstrates strong interdependencies among the three pillars, each reinforcing and sustaining the others, the integration of AI at the institutional level introduces a novel and promising dimension for both research and theory development.
